# Prognostic significance of tumor infiltrating lymphocytes on first-line pembrolizumab efficacy in advanced non-small cell lung cancer

**DOI:** 10.1007/s12672-023-00615-4

**Published:** 2023-01-20

**Authors:** Kyoichi Kaira, Ou Yamaguchi, Tomonori Kawasaki, Kousuke Hashimoto, Yu Miura, Ayako Shiono, Atsuto Mouri, Hisao Imai, Kunihiko Kobayashi, Masanori Yasuda, Hiroshi Kagamu

**Affiliations:** 1 Department of Respiratory Medicine, Comprehensive Cancer Center, International Medical Center, Saitama Medical University, 1397-1 Yamane, Hidaka-City, Saitama 350-1298 Japan; 2 Department of Pathology, International Medical Center, Saitama Medical University, 1397-1 Yamane, Hidaka-City, Saitama 350-1298 Japan

**Keywords:** Tumor-infiltrating lymphocytes, CD4, CD8, PD-1, PD-L1, Non-small cell lung cancer

## Abstract

**Aim:**

Tumor-infiltrating lymphocytes (TILs) in the tumor and stroma are expected to accurately predict the efficacy of programmed death-1 (PD-1) blockade therapy. However, little is known about the prognostic significance of TILs in first-line PD-1 therapy. We assessed TILs in patients with advanced or metastatic non-small cell lung cancer (NSCLC) treated with pembrolizumab in the palliative setting.

**Methods:**

Multiplex immunohistochemistry staining of TILs (CD4, CD8, Foxp3, and PD-1) and immunohistochemical staining of CK and PD-L1 in the tumor and stroma was performed in tumor specimens of 107 NSCLC patients and correlated with clinical outcomes, as a single-center retrospective study. TILs and programmed death ligand-1 (PD-L1) were assessed on biopsies (N = 93) or surgical resections (N = 14) before first-line pembrolizumab.

**Results:**

A low number of stromal CD4 TILs were significantly associated with bone metastasis and poor performance status (PS). The median progression-free survival (PFS) and overall survival (OS) were significantly higher in patients with a high number of stromal CD4 TILs (336 days and 731 days, respectively) than in those with low infiltration (204 days and 333 days, respectively). Patients with a high number of intratumoral CD8 TILs (731 days) yielded significantly better OS than those with low infiltration (333 days), but not for PFS. Multivariate analysis confirmed that stromal CD4 TILs were independent predictors of PFS, but not OS. Furthermore, intratumoral CD8 TILs were independent predictors of better OS. In the survival analysis of key subgroups, stromal CD4 TILs were identified as significant predictors of survival in patients with non-adenocarcinomatous histology and PD-L1 ≥ 50%.

**Conclusion:**

Stromal CD4 TILs were identified as a significant marker for predicting the PFS after pembrolizumab therapy, especially in patients with non-adenocarcinoma and high PD-L1 expression. In addition, intratumoral CD8 TILs were identified as significant predictors of OS.

**Supplementary Information:**

The online version contains supplementary material available at 10.1007/s12672-023-00615-4.

## Introduction

Non-small cell lung cancer (NSCLC) is the most common cancer type and a leading cause of cancer-related deaths worldwide. Currently, immune checkpoint inhibitors (ICIs) such as programmed death-1 (PD-1)/programmed death ligand-1 (PD-L1) antibodies are standards of care for advanced NSCLC with genomic alteration. To predict the efficacy of PD-1 blockade, PD-L1 expression on tumor cells is widely used. However, its predictive significance is limited, since some patients do not respond to PD-1 blockade based on the expression of PD-L1 [1]. Meanwhile, the presence of tumor-infiltrating lymphocytes (TILs) in the tumor and stromal compartments is expected to accurately predict the efficacy of PD-1 blockade. However, only a few studies with small sample sizes studied the clinical significance of TILs as predictive markers for patients with advanced NSCLC receiving PD-1 blockade [2–4].

Recently, Hashemi et al. reported the prognostic significance of stromal TILs as a powerful predictor of PD-1 blockade initiation in 141 patients with previously treated NSCLC [2]. In their study, only CD8 + TILs were immunohistochemically examined on the tumor and stroma, being stromal CD8 + TILs identified as the strongest predictors of progression-free survival (PFS) and overall survival (OS) on anti-PD-1 therapy [2]. A preliminary study of 38 patients with advanced NSCLC treated using pembrolizumab (21% in first line and 79% in subsequent treatments) showed that CD8 TILs significantly correlated with objective response rate (ORR) and PFS. However, CD4 TILs was not associated with these outcomes [3]. Additionally, one study examined the therapeutic significance of CD4 + and CD8 + TILs as predictive markers for response to PD-1 blockade as any treatment line in 26 patients with advanced NSCLC [4]. Although no statistically significant relationship between CD4 + lymphocytes and ORR to PD-1 blockade was recognized, the tumors with CD8 + lymphocyte count < 886/mm^2^ displayed a low response rate. Conversely, the ORR was high when CD8 + lymphocyte count ranged from 886 to 1889/mm [2, 4]. These studies described that the assessment of TILs was performed using the samples of biopsies or resections prior to the start of ICI [1–4]. All previous studies included the NSCLC patients treated with PD-1 blockade monotherapy as second or more lines, therefore, the tumor immune environment in the tumor specimens used for immunohistochemical staining may be affected by chemotherapeutic agents prior to PD-1 blockade administration [2–4]. Conventional immunohistochemistry was performed in previous studies as a pathological technique for evaluating TILs. However, it is time consuming to accurately count the number of TILs in the tumor and stroma compartments using conventional immunohistochemistry and the pathologists eye. The development of more accurate quantitative device is warranted to count TILs in the different compartments. As such, further studies should focus on the prognostic significance of TILs in the tumor tissues of NSCLC patients receiving first-line anti-PD-1 monotherapy with sufficient sample sizes to elucidate the role of TILs as therapeutic predictors of TILs. Recently, Kagamu et al. reported that the CD4^+^ T cell meta-cluster in peripheral blood just before PD-1 blockade therapy could predict the outcome of advanced NSCLC patients after its treatment, moreover, correlated with CD4^+^ T cell infiltration in the tumor microenvironment, moreover, peripheral Th1 correlated with CD8^+^ T cell infiltration [5].

As such, we conducted a clinicopathologic study to elucidate the prognostic significance of TILs in patients with advanced NSCLC who received first-line pembrolizumab monotherapy. We used multiplex immunofluorescence (IF) imaging to accurately count the number of TILs on the tumor and stroma components separately by immunohistochemistry.

## Methods

### Patients

One hundred and seventy-nine patients with advanced or metastatic NSCLC without any driver mutations received first-line pembrolizumab monotherapy at our institution from March 2017 to March 2021. Among them, 72 did not have primary tumor specimens just before first-line pembrolizumab treatment. Of these 72 patients, 13 had sampling error for the assessment of TILs because of small tumor specimens by biopsy and 59 displayed no biopsy sample due to prior examination for any driver mutations. Therefore, 107 patients (median age of 71 years; 93 men and 16 women) were included in this study. Clinical data were extracted from medical records. TILs and PD-L1 were assessed on biopsies (N = 93) or surgical resections (N = 14) before first-line pembrolizumab. The sample of the current study is overlapping to that of previously reported approach [5]. This study was approved by the Institutional Ethics Committee of the International Medical Center, Saitama Medical University. The requirement for written informed consent was waived by the ethics committee of Saitama Medical University because of the retrospective nature of the study.

### Treatment and evaluation

For first-line monotherapy, 200 mg/day pembrolizumab was intravenously administered. Physical examination, complete blood count (CBC), biochemical testing, and adverse events were measured according to the judgement of each chief physician. Any toxicity was graded based on the Common Terminology Criteria for Adverse Events version 4.0. Tumor response was examined according to the response evaluation criteria in solid tumors (RECIST) version 1.1 [6].

### Specimen characteristics

Immunohistochemical assessment was performed using 107 primary sites of NSCLC before initiating first-line pembrolizumab treatment. Of 107 patients, 14 (13.1%) were immunohistochemically examined by surgical specimens and 93 (86.9%) by biopsy specimens.

### Study design

This is retrospective study to evaluate the clinicopathologic significance of TILs in patients with advanced NSCLC who received pembrolizumab monotherapy as first-line setting by immunohistochemistry. The clinical endpoints of our study examined the prognostic value of CD4, CD8 and Foxp3 in stroma and intratumoral lesions correlated with the efficacy and outcome of pembrolizumab. Aside from these TILs, PD-1 and PD-L1 were also examined by immunohistochemistry.

### Multiplex immunohistochemistry (OPAL™) staining, image acquisition and data analysis

The tumor specimens were formalin-fixed and paraffin-embedded (FFPE). Then, three sections with the largest area of viable tumor cells were selected. Afterwards, 5-μm-thick sections of FFPE tissue were deparaffinized and rehydrated using xylene and ethanol for multiplex immunohistochemistry (mIHC) staining (fixative concentration, 10%; temperature, room; duration 30 min). Next, all slides were treated with 0.3% hydrogen peroxide in methanol for 30 min to block endogenous peroxidase activity. To expose antigens, sections were autoclaved in 10 mmol l^−1^ sodium citrate buffer (pH 6.0) for 20 min, followed by microwave treatment at 98 °C for 15 min, and cooled for 30 min. After rinsing in 0.05 M tris-buffered saline containing 0.1% Tween 20, the sections were incubated with mouse monoclonal CD4 (Leica Biosystems clone 4B12, 1/100, high pH retrieval), mouse monoclonal CD8 (DAKO, clone C8/144B 1/150 high pH retrieval), mouse monoclonal FOXP3 (Abcam, 1/50, clone 236A/E7, pH6 retrieval), mouse monoclonal PD-L1 (IHC 22C3 PharmaDx, Dako), mouse monoclonal PD-1 (Abcam,1/100,cloneNAT105,pH6retrieval) and mouse monoclonal pan cytokeratin (Abcam clone AE1/AE3 1/100 pH6). Blocking reagent (1:10; Antibody Diluent, PerkinElmer) was incubated at room temperature, 10 min. Secondary antibody (1:10; dilution; 2 drops of Opal Polymer HRP, PerkinElmer) (cat. NEL744001KT) was incubated for room temperature. 10 min. using Akoya: NEL 81001KT opal-7-color Manual IHC KIT (opal Polymer HRP MS + Rb). Chromogen detection reagent for HRP/DAB was Akoya: NEL 811001KT opal-7-color Manual IHC KIT (1Xplus Amplification Diluent) and counterstain was incubated for room temperature, 5 min. using Spectral DAPI solution.

Immunofluorescence signals were visualized using the OPAL™ 7-color IHC kit (akoya biosciences, MA) TSA dyes 520, 540, 570, 620, 650, and 690, counterstained with Spectral DAPI. All slides were imaged on the Mantra 2 Quantitave Pathology Workstation (akoya biosciences MA). Color separation, tissue and cell segmentation, and cell phenotyping were performed using inForm^®^ Sofware v2.5.1 (akoya bioscience MA) to extract image data. Slides were evaluated for the presence of TILs within the tumor and stroma. mIHC staining and data analysis were performed according to previously described procedures [7].

### Multiplex IHC data analysis

All slides were scanned at 20 × magnification to achieve high-powered imaging at a resolution of 0.5 µm per pixel using Phenochart (akoya biosciences MA). High-powered imaging included intratumoral areas with lymphocytic infiltrates, and stromal areas. An algorithm was designed based on pattern recognition of pancytokeratin-positive areas (tumor) and pancytokeratin-negative areas (stroma). Cell segmentation was performed on all cells counter-stained with DAPI. The TIL scoring for its distribution was performed on the 20 × pre-scanned images of each patient. Three high-powered images within the tumor parenchyma and stroma with the highest TIL density were selected to grade TIL density along with OPAL TIL count and percentage. Multiple images (3 images) from the tumor and stroma were quantified. The cell count of TILs was determined by normalizing to 1000 cells after counting all cells Images were analyzed on inForm 2.5.1 software (Akoya Biosciences, MA). Type of microscope was Mantra2 multispectral microscopy (Akoya Biosciences, Marlborough, MA) with magnification: 20 × objective. Fluorescence images were acquired on Mantra2 multispectral microscopy (Akoya Biosciences, Marlborough, MA) with 20 × objective.

### Statistical analysis

For statistical analyses, we used Student’s *t-test* and the χ^2^ test for continuous and categorical variables, respectively. The statistical significance level was set at *P* < 0.05. Correlations between TIL measurements and variables were analyzed using the Pearson’s rank test. Tumor PD-L1 expression was counted as tumor proportional score (TPS), and classified into 2 categories of 1–49% and 50–100%. The cut-off value of TILs in the tumor and stroma was defined as the approximate value of the median counts. PFS was defined as the time from the initial pembrolizumab treatment to disease progression or death. OS was defined as the time from the initial pembrolizumab to death from any cause. The Kaplan–Meier method was used to estimate survival as a function of time, and survival differences were analyzed using the log-rank test. Univariate and multivariate analyses according to different variables were performed using logistic regression analysis. All statistical analyses were performed using GraphPad Prism software (v.8.0; GraphPad Software, San Diego, CA, USA) and JMP 14.0 (SAS Institute Inc., Cary, North Carolina, USA).

## Results

### Patient demographics

A total of 107 patients were analyzed whose characteristics are summarized in Table 1. Adenocarcinoma (AC) and squamous cell carcinoma were observed in 52 (45.6%) and 45 (42.1%) patients, respectively. In the histological types of “Other”, sarcomatoid carcinomas was observed in 3 patients, adenosquamous carcinoma in 3 patients, and large cell carcinoma in 4 patients. Twenty-six (24.2%) patients experienced grade 3 or 4 immune-related adverse events (irAEs). The median follow-up period was 364 days.

**Table 1:** Patient’s characteristics

Different variables		N = 107 (%)
Age	< 75/ ≥ 75 years	64/43 (59.8/40.2)
Gender	Male/Female	91/16 (85.0/15.0)
ECOG PS	0/1/2/3	31/51/16/9 (28.9/47.7/15.0/8.4)
Smoking history	Yes/No	96/11 (89.7/10.3)
Histological types	AC/SQC/Other	52/45/10 (45.6/42.1/9.3)
Disease staging	III/IV/Ope rec./CRT rec	11/77/17/2 (10.2/72.0/15.9/1.9)
Metastatic sites	Brain yes/no Liver yes/no Bone yes/no	30/77 (28.0/72.0) 9/98 (8.4/91.6) 25/82 (23.4/76.6)
Tumor response	CR/PR/SD/PD/NE	0/39/40/23/5 (0/36.4/37.4/21.6/4.6)
PD-L1 (TPS) (%)	1 – 49/50–100	26/81 (24.3/75.7)
Radiation before initial pembrolizumab	Yes/No	38/69 (35.5/64.5)
Grade 3/4 irAE	Yes/No	26/81 (24.2/75.8)
Lymphocyte	Low/High (< 1223/ ≥ 1223)	53/54 (49.5/50.5)
Albumin	Low/High (< 3.5/ ≥ 3.5)	55/52 (51.4/48.6)
CRP	Low/High (< 1.0/ ≥ 1.0)	54/53 (50.5/49.5)

*ECOG* eastern cooperative oncology group, *PS* performance status, *PD-L1* programmed death ligand-1, *TPS* tumor proportional score, *irAE* immune-related adverse events, *AC* adenocarcinoma, *SQC* squamous cell carcinoma, *Other* other histology except for AC and SQC, *Ope rec.*, recurrence after operation, *CRT rec.* recurrence after chemoradiotherapy, *CR* complete response, *PR* partial response, *SD* stable disease, *PD* progressive disease, *NE* not evaluable, *CRP* C-reactive protein

The cut-off value of lymphocyte, albumin and CRP was defined as the value of median counts

### Immunohistochemical findings and clinical efficacy

TPS was counted as the estimated percentage (0–100%) of tumor cells with partial or complete membranous PD-L1 immunostaining, and 26 (24.3%) patients exhibited a TPS of 1–49% and 81 (75.7%) patients displayed a TPS of 50–100% (Table 1). The 101 patients with evaluable targeting lesions achieved an ORR of 34.6% and a disease control rate (DCR) of 76.2%. The ORR in patients with PD-L1 expression of 1–49% and 50–100% was 25.0% (7/28 patients) and 39.5% (32/81 patients), respectively (*p* = 0.25). In the 13 patients who had sampling error, the assessment for TILs analysis by immunohistochemistry was impossible due to inadequate tumor specimen after cutting.

**Fig. 1:**
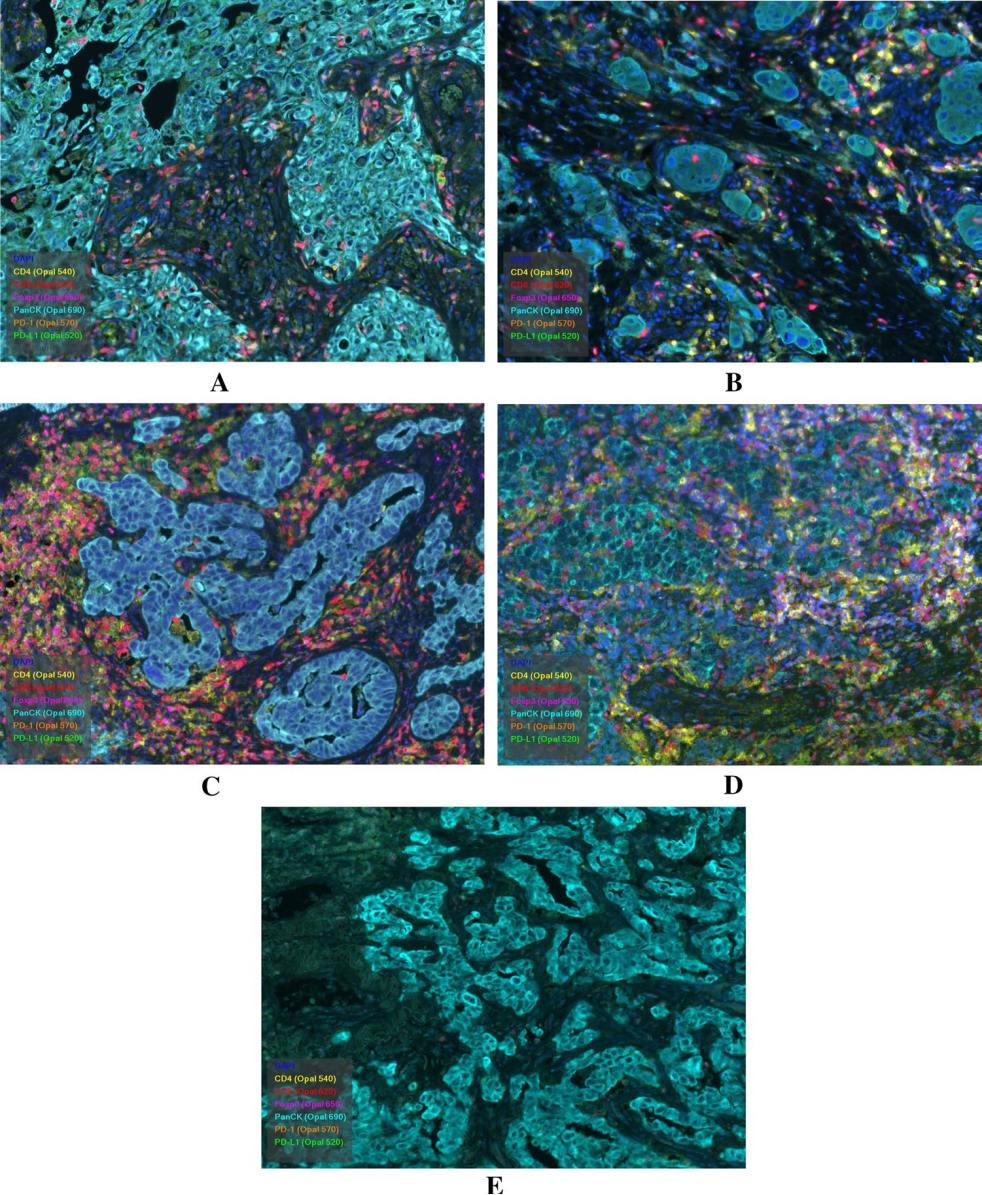
CD8, Foxp3 and PD-1 lymphocytes were observed in the stroma and tumor (**A**). There was evidence of stromal infiltration of CD4 and CD8 lymphocytes (**B**). CD4, CD8, and Foxp3 lymphocytes highly infiltrated the stroma (**C**). Intratumoral infiltration of CD4, CD8, Foxp3, and PD-1 lymphocytes is highly recognized (**D**). There was no evidence of intratumoral or stromal infiltration of lymphocytes (**E**)

The cell counts of individual TILs were independently performed between the intratumoral and stromal area. In the tumor, the median values of individual TILs were 8.0 (0–589) for CD4, 12.1 (0–583) for CD8, 7.1 (0–658) for Foxp3 and 0.1 (0–151) for PD-1. In the stroma, the values were 17.1 (0–442) for CD4, 34.4 (0–739) for CD8, 8.7 (0–205) for Foxp3, and 0.1 (0–286) for PD-1. As approximate values of median TILs, the cut-off values in intratumoral TILs were defined as 10 for CD4, CD8, 10 for Foxp3 and 1.0 for PD-1. Meanwhile, the stromal TIL values were 20 for CD4, 30 for CD8, 10 for Foxp3, and 1.0 for PD-1. Representative multiplex ID staining is shown in Fig. 1. The numbers of intratumoral and stromal CD4, CD8, Foxp3, and PD-1 TILs were not significantly different to PD-L1 expression of 1–49% and 50–100% (Additional file 1: Fig S1, online only), as well as between partial response (PR) and non-PR (Additional file 2: Fig. S2, online only). Next, the patients’ demographics based on the cut-off value of individual TILs in the tumor and stroma were compared (Additional file 3: Table S1 and Additional file 4: Table S2, online only). A high number of intratumoral and stromal Foxp3 TILs were significantly associated with adenocarcinoma. Moreover, stromal Foxp3 TILs was significantly associated with tumor response, history of prior radiotherapy, and grade 3/4 immune-related adverse events (irAEs). The low number of intratumoral and stromal PD-1 TILs was significantly associated with bone metastasis. Additionally, the low number of stromal CD4 TILs was significantly linked to bone metastasis and poor PS (Additional file 4: Table S2, online only). Next, the mutual correlation between intratumoral/stromal TILs (CD4, CD8, Foxp3, PD-1) was analyzed using the Pearson’s rank test (Additional file 5: Table S3, online only). The numbers of intratumorl CD4, CD8, Foxp3 and, PD-1 significantly correlated with those of stromal CD4 (γ = 0.624, *p* < 0.001), CD8 (γ = 0.741, *p* < 0.001), Foxp3 (γ = 0.386, *p* < 0.001), and, PD-1 (γ = 0.907, *p* < 0.001), respectively. Moreover, there was also a statistically significant correlation between intratumoral CD4 and CD8 (γ = 0.247, *p* = 0.009), between stromal CD4 and CD8 (γ = 0.209, *p* = 0.029), between intratumoral Foxp3 and stromal CD8 (γ = 0.216, *p* = 0.023), and between stromal CD8 and Foxp3 (γ = 0.287, *p* = 0.002).

### Univariate and multivariate survival analysis

**Fig. 2:**
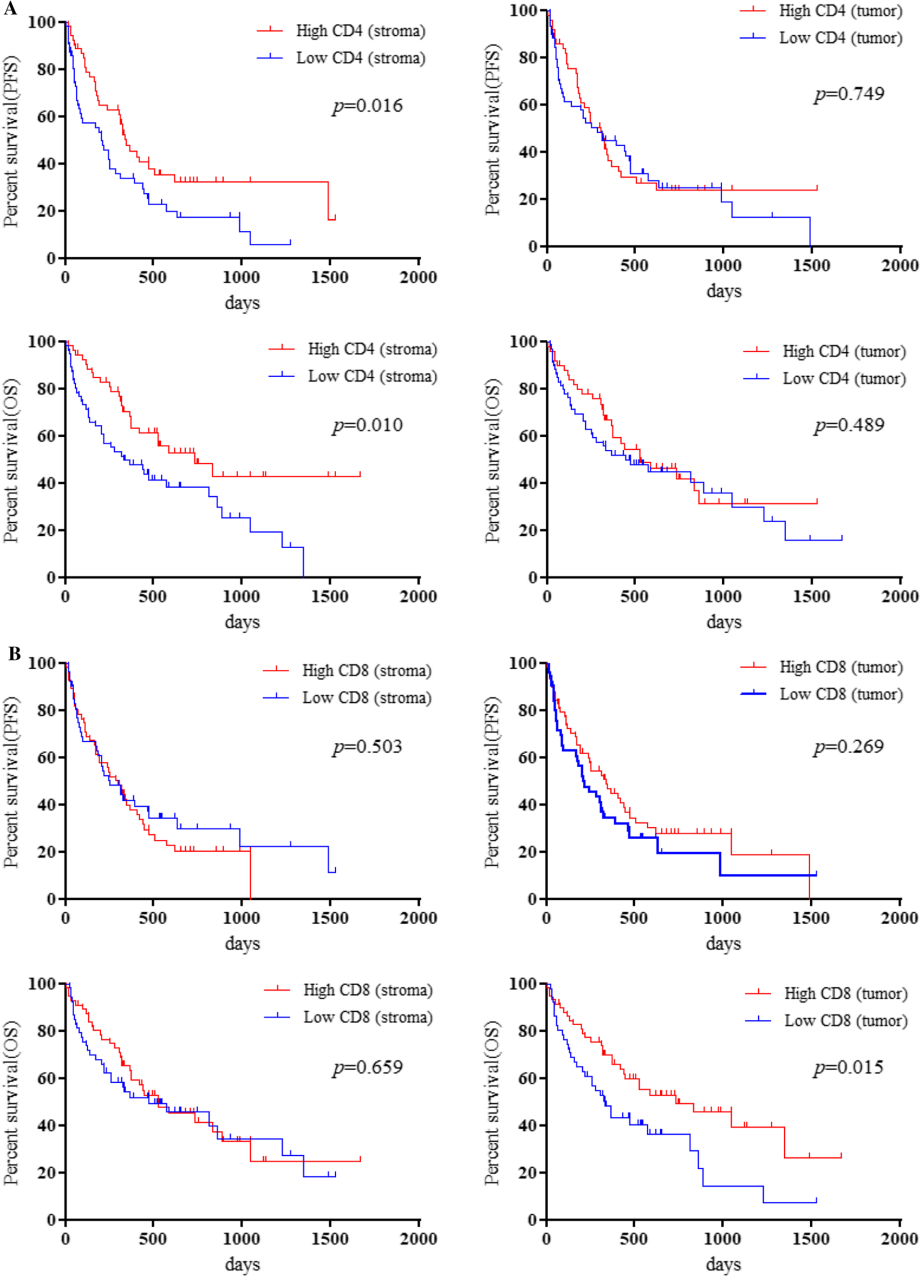

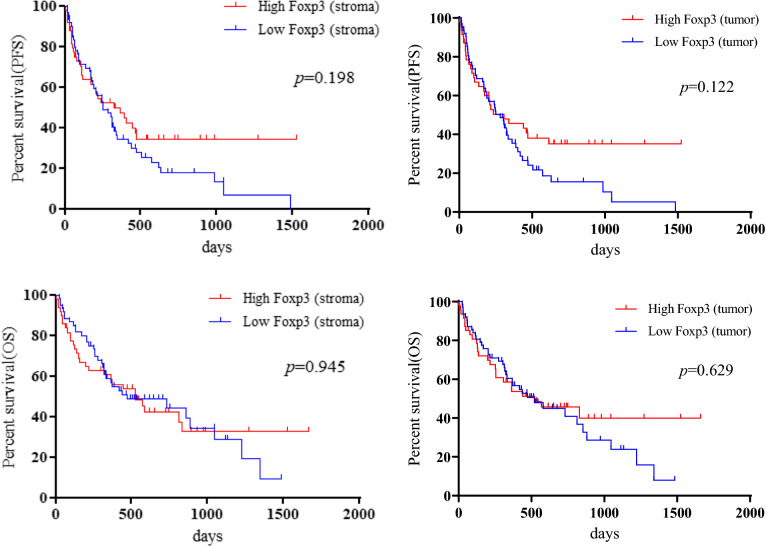
Kaplan–Meier curves in the PFS and OS according to numbers of TILs in tumor and stroma. Patients with high stromal CD4 TILs showed a significantly better PFS (*p* = 0.016) and OS (*p* = 0.010) than those with low stromal CD4 TILs, whereas there was no significant difference in PFS and OS between patients with high and low intratumoral CD4 TILs (**A**). High intratumoral CD8 TILs could significantly predict better OS after pembrolizumab administration, but not PFS. Moreover, there was no statistically significant difference in the PFS and OS according to the level of stromal CD8 TILs, stromal Foxp3 TILs, and intratumoral Foxp3 TILs observed (**B**, **C**)

The median PFS and OS were 251 and 521 days, respectively. Eighty (74.7%) of the 107 patients experienced recurrence, and 66 (61.7%) patients died due to disease progression. Kaplan–Meier curves for PFS and OS according to TILs in the tumor and stroma are shown in Fig. 2. Univariate and multivariate analyses were performed in all patients (Table 2). Univariate analysis identified histology and stromal CD4 TILs as significant prognostic factors for PFS, and PS, C-reactive protein (CRP), albumin, intratumoral CD8 TILs, and stromal CD4 TILs as significant predictors of OS. Application of a univariate log-rank test enabled the screening of variables with a cut-off of *p* < 0.10 for subsequent multivariate analysis. Multivariate analysis confirmed that histology and stromal CD4 TILs were independent prognostic factors for predicting better PFS. Meanwhile, PS and intratumoral CD8 TILs were identified as independent factors for predicting favorable OS (Table 2). Survival analysis of key subgroups based on the level of stromal CD4 TILs and intratumoral CD8 TILs was conducted (Tables 3, 4). We found that a high number of stromal CD4 TILs was significantly associated with favorable outcomes in patients the following characteristics: < 75 years, males, heavy smokers, non-ACs, without brain metastases, non-PR, with low lymphocytes, and PD-L1 ≥ 50% (Table 3). Meanwhile, < 75 years, PS of 2–3, AC, brain metastasis, prior radiotherapy, high albumin, low lymphocytes, and PD-L1 1–50% were identified as significant factors for OS but not PFS, in intratumoral CD8 TILs (Table 4).

**Table 2:** Univariate and multivariate survival analysis in all patients (n = 107)

Different variables			Progression-free survival						Overall survival				
			Univariate analysis		Multivariate analysis				Univariate analysis		Multivariate analysis		
		MST	HR (95% CI)	*p*-value	HR	95% CI	*p*-value	MST	HR (95% CI)	*p*-value	HR	95% CI	*p*-value
Age	< 75/ ≥ 75 years	324/251	1.14 (0.73–1.80)	0.55				441/568	0.98 (0.60–1.61)	0.92			
Gender	Male/female	281/270	0.99 (0.54–1.80)	0.94				418/829	1.62 (0.84–3.13)	0.22			
ECOG PS	0–1/2–3	324/209	2.03 (1.13–3.67)	0.09	1.28	0.94–1.74	0.11	731/169	2.75 (1.47–5.14)	** < 0.01**	1.59	1.14–2.21	** < 0.01**
Smoking (BI)	< 900/ ≥ 900	234/324	0.79 (0.51–1.23)	0.33				521/568	0.94 (0.58–1.53)	0.84			
Histology	AC/Non-AC	388/244	1.68 (1.08–2.61)	**0.01**	0.73	0.56–0.94	** < 0.01**	813/372	1.42 (0.88–2.31)	0.06	0.83	0.63–1.08	0.16
Brain metastasis	Yes/No	470/234	1.30 (0.81–2.07)	0.18				522/437	1.26 (0.75–2.13)	0.22			
Bone metastasis	Yes/No	244/307	1.21 (0.71–2.06)	0.40				522/521	1.05 (0.60–1/83)	0.64			
Prior RT	Yes/No	324/251	1.08 (0.67–1.75)	0.97				472/581	1.16 (0.69–1.93)	0.83			
CRP	High/Low	209/324	1.51 (0.96–2.36)	0.09	0.97	0.74–1.25	0.86	364/731	1.79 (1.10–2.91)	**0.02**	1.01	0.73–1.39	0.93
Albumin	High/Low	303/251	1.51 (0.97–2.35)	0.08	0.96	0.74–1.25	0.79	829/364	1.84 (1.13–2.98)	**0.01**	0.88	0.65–1.19	0.42
Lymphocyte	High/Low	251/281	1.02 (0.66–1.58)	0.91				441/521	1.07 (0.66–1.73)	0.95			
PD-L1 expression	1–49/50–100%	240/324	1.01 (0.60–169)	0.82				441/521	0.97 (0.55–1.72)	0.99			
Intratumoral PD-1	High/Low	234/303	1.27 (0.74–2.15)	0.63				372/568	1.38 (0.77–2.46)	0.22			
Intratumoral CD4	High/Low	303/281	1.07 (0.68–1.68)	0.74				522/441	1.19 (0.72–1.95)	0.48			
Intratumoral CD8	High/Low	336/209	1.28 (0.81–2.02)	0.26				731/333	1.81 (1.09–3.01)	**0.01**	0.70	0.53–0.92	**0.01**
Intratumoral Foxp3	High/Low	307/281	1.43 (0.91–2.24)	0.12				522/472	1.13 (0.68–1.86)	0.62			
Stromal PD-1	High/Low	234/307	1.17 (0.71–1.93)	0.24				372/568	1.21 (0.71–2.05)	0.41			
Stromal CD4	High/Low	336/204	1.72 (1.09–2.69)	**0.01**	0.79	0.62–0.99	**0.04**	731/333	1.92 (1.16–3.16)	**0.01**	0.89	0.67–1.18	0.44
Stromal CD8	High/Low	303/251	0.85 (0.54–1.34)	0.50				521/472	1.16 (0.68–1.83)	0.65			
Stromal Foxp3	High/Low	324/251	1.34 (0.86–2.11)	0.19				522/472	0.98 (0.59–1.61)	0.94			

Bold values indicate statistically significant *p* values (*p* < 0.05)

*MST* median survival time (days), *ECOG PS* eastern cooperative oncology group performance status, *BI* brinkman index, *AC* adenocarcinoma, *non-AC* non-adenocarcinoma, *prior RT* radiation before initial pembrolizumab, *CRP* C-reactive protein, *PD-L1* programmed death ligand-1, *PD-1* programmed death-1, *HR* hazard ratio, *95% CI* 95% confidence interval

**Table 3:** Survival analysis of subgroups according to stromal CD4 TILs

Different variables		Number of cases (n = 107)	Progression-free survival		Overall survival	
			Hazard ratio (95% CI)	p-value	Hazard ratio (95% CI)	p-value
Age	< 75 years	64	0.553 (0.304–1.007)	**0.044**	0.443 (0.235–0.833)	**0.018**
	≥ 75 years	43	0.646 (0.326–1.281)	0.209	0.675 (0.303–1.503)	0.338
Gender	Male	91	0.529 (0.322–0.870)	**0.009**	0.556 (0.327–0.945)	**0.030**
	Female	16	1.050 (0.351–3.131)	0.927	0.381 (0.086–1.677)	0.181
Smoking	BI < 900	54	0.725 (0.391–1.345)	0.291	0.527 (0.257–1.080)	0.061
	BI ≥ 900	53	0.491 (0.255–0.945)	**0.032**	0.452 (0.223–0.915)	**0.038**
PS	PS = 0–1	82	0.605 (0.362–1.039)	0.055	0.595 (0.315–1.121)	0.096
	PS = 2–3	25	0.828 (0.317–2.161)	0.707	0.849 (0.329–2.192)	0.108
Histology	AC	52	0.785 (0.398–1.545)	0.472	0.553 (0.261–1.170)	0.113
	Non-AC	55	0.455 (0.252–0.821)	**0.007**	0.571 (0.299–1.089)	0.093
Brain meta	Yes	30	0.542 (0.216–1.357)	0.155	0.365 (0.126–1.051)	**0.044**
	No	77	0.660 (0.388–1.120)	0.127	0.646 (0.362–1.152)	0.141
Bone meta	Yes	25	0.744 (0.288–1.921)	0.562	0.739 (0.260–2.099)	0.586
	No	82	0.553 (0.325–0.943)	**0.021**	0.477 (0.266–0.858)	**0.010**
ORR	PR	39	0.733 (0.343–1.567)	0.403	0.851 (0.328–2.204)	0.112
	Non-PR	63	0.520 (0.301–0.900)	**0.017**	0.465 (0.263–0.821)	**0.009**
Prior RT	Yes	38	0.589 (0.268–1.292)	0.176	0.223 (0.095–0.518)	**0.001**
	No	69	0.603 (0.351–0.038)	0.062	0.803 (0.438–1.471)	0.476
CRP	High	53	0.628 (0.337–1.168)	0.132	0.593 (0.311–1.131)	0.112
	Low	54	0.571 (0.299–1.088)	0.076	0.496 (0.231–1.067)	0.066
Albumin	High	52	0.675 (0.346–1.314)	0.226	0.668 (0.308–1.451)	0.301
	Low	55	0.544 (0.297–0.997)	0.053	0.485 (0.256–0.916)	**0.030**
Lymphocyte	High	54	0.717 (0.381–1.353)	0.286	0.651 (0.315–1.345)	0.224
	Low	53	0.464 (0.244–0.886)	**0.021**	0.409 (0.204–0.822)	**0.022**
PD-L1	1–49%	26	0.685 (0.278–1.684)	0.406	0.742 (0.278–1.977)	0.545
	50–100%	81	0.580 (0.348–0.967)	**0.032**	0.476 (0.271–0.836)	**0.015**

Bold values indicate statistically significant *p* values (*p* < 0.05)

**Table 4:** Survival analysis of subgroups according to intratumoral CD8 TILs

Different variables		Number of cases (n = 107)	Progression-free survival		Overall survival	
			Hazard ratio (95% CI)	p–value	Hazard ratio (95% CI)	p–value
Age	< 75 years	64	0.611 (0.325–1.149)	0.089	0.358 (0.183–0.702)	** < 0.001**
	≥ 75 years	43	1.261 (0.641–2.481)	0.487	1.412 (0.640–3.114)	0.377
Gender	Male	91	0.904 (0.556–1.468)	0.679	0.646 (0.385–1.084)	0.088
	Female	16	0.432 (0.127–1.469)	0.099	0.331 (0.052–2.120)	0.086
Smoking	BI < 900	54	1.012 (0.551–1.858)	0.968	0.591 (0.293–1.192)	0.115
	BI ≥ 900	53	0.738 (0.383–1.425)	0.346	0.584 (0.289–0.179)	0.113
PS	PS = 0–1	82	0.893 (0.528–1.509)	0.668	0.663 (0.355–1.235)	0.182
	PS = 2–3	25	0.503 (0.203–1.252)	0.066	0.438 (0.189–1.014)	**0.021**
Histology	AC	52	0.839 (0.425–1.658)	0.605	0.475 (0.223–1.010)	**0.039**
	Non-AC	55	0.799 (0.442–1.448)	0.448	0.733 (0.381–1.413)	0.334
Brain meta	Yes	30	0.457 (0.172–1.215)	0.051	0.381 (0.127–1.137)	**0.031**
	No	77	1.008 (0.601–1.688)	0.977	0.725 (0.413–1.273)	0.257
Bone meta	Yes	25	0.734 (0.305–1.768)	0.486	0.483 (0.184–1.270)	0.112
	No	82	0.855 (0.507–1.441)	0.547	0.652 (0.366–1.158)	0.128
ORR	PR	39	0.752 (0.339–1.666)	0.580	0.646 (0.232–1.805)	0.347
	Non-PR	63	0.876 (0.509–1.507)	0.619	0.595 (0.338–1.050)	0.064
Prior RT	Yes	38	0.714 (0.333–1.528)	0.833	0.411 (0.182–0.928)	**0.026**
	No	69	0.955 (0.552–1.654)	0.858	0.771 (0.415–1.435)	0.396
CRP	High	53	0.863 (0.464–1.602)	0.625	0.622 (0.325–1.188)	0.119
	Low	54	0.813 (0.426–1.549)	0.518	0.598 (0.278–1.286)	0.167
Albumin	High	52	0.679 (0.348–1.327)	0.232	0.448 (0.204–0.984)	**0.032**
	Low	55	0.980 (0.536–1.791)	0.947	0.815 (0.433–1.534)	0.515
Lymphocyte	High	54	0.848 (0.448–1.605)	0.603	0.747 (0.361–1.547)	0.408
	Low	53	0.791 (0.418–1.495)	0.458	0.455 (0.229–0.905)	**0.021**
PD-L1	1–49%	26	0.842 (0.508–1.394)	0.496	0.555 (0.317–0.971)	**0.034**
	50–100%	81	0.771 (0.277–2.143)	0.590	0.745 (0.241–2.300)	0.575

Bold values indicate statistically significant *p* values (*p* < 0.05)

## Discussion

This is a clinicopathological study to elucidate the prognostic significance of TILs in the tumor and stroma of patients with advanced NSCLC receiving first-line pembrolizumab monotherapy. We found that stromal CD4 TILs could be viable biomarkers for predicting the outcome of first-line PD-1 blockade. Additionally, intratumoral CD8 TILs were identified as an independent predictor for OS. Compared to previous studies, the immunofluorescent visualization used in our study could exactly count the number of TILs in the tumor and stroma compartments. Regarding the mutual correlation between TILs tumor/stroma, the numbers of individual TILs in the intratumoral lesions exhibited a similar trend to those in the stromal lesions. In particular, a significant correlation between the numbers of CD4 and CD8 TILs in tumor/stroma was observed, but, those of stromal CD8 TILs correlated with Foxp3. The cooperative infiltration of CD4 in stromal with CD8 in intratumoral lesions may play a crucial role in the prognostic significance after PD-1 blockade treatment, suggesting an overall same prognostic value of CD4 and CD8.

Recently, there have been several reports regarding the relationship between TILs and prognosis in patients with NSCLC. A meta-analysis of 8600 patients with NSCLC showed that CD8 TILs were closely linked to favorable outcomes. Meanwhile, CD4 TILs were identified as significant predictors when evaluated in tumor stroma rather than tumor cell nests [8]. In a recent review, Wei et al. suggested that CD8 TILs mediate stronger antitumor responses than CD4 TILs [9]. However, previous reports didn’t focus on NSCLC patients who received immunotherapy as 2nd or more over lines. Moreover, the numbers of TILs were manually counted [2, 10], and archival tumor specimens before chemotherapy followed by immunotherapy were used for TIL assessment; therefore, the immune environment for TILs may be affected by chemotherapy.

By a recent meta-analysis, first-line ICIs in NSCLC patients with poor PS were described to be associated with worse outcome [11, 12]. As our study indicated that low numbers of stromal CD4 TILs was closely related to poor PS, the degree of CD4^+^ T cell infiltration into the stroma may affect the patient’s general condition. Furthermore, survival analysis according to histology revealed that the predictive role of intratumoral CD8 TILs was recognized in AC patients, whereas stromal CD4 TILs were identified as prognostic factors for patients with non-AC. However, it remains unclear whether the distribution and characteristics of TILs differ according to histological type. Several studies unrelated to ICI therapy reported that CD8 + T cell infiltration was closely associated with improved outcomes in lung cancer [13–15]. However, the tumor immune microenvironment may be affected by immunotherapy; thus, it is difficult to determine the prognostic significance of CD8 TILs as biomarkers. Recently, CD4 + T cells were demonstrated to be responsible for T-cell regulation and cytokine production; thus, the role of CD4^+^ T cells is focused on the effect of T cell responses against tumors. Kagamu et al*.* reported that a higher number of CD62L^low^ CD4^+^ T cells prior to PD-1 blockade therapy in the peripheral blood of previously treated NSCLC patients could predict long-term response to treatment [5, 16]. Moreover, Inomata et al. also reported that high numbers of CD4^+^ PD1^+^ T cells and CD62L^low^ CD4^+^ T cells in the peripheral blood were could predict longer response to PD-1 blockade in NSCLC patients [16, 17]. Recently, high stromal infiltration of CD4 and CD8 in tumor specimens were reported to predict the response to nivolumab treatment in previously treated NSCLC compared to those with low infiltration [18]. A recent meta-analysis demonstrated that stromal infiltration of CD4^+^ T cells is closely linked to a favorable prognosis in NSCLC [19]. In the present study, CD4 in stroma was significant predictors for the patients with PD-L1 ≥ 50%, whereas, CD8 in intratumoral lesions for those with PD-L1 1–49%. Although the different function of TILs according to PD-L1 expression may be novel evidence, it remains unknown about the prognostic relationship between different TILs and PD-L1 expression after first-line PD-1 blockade.

Our study had several limitations. First, most of our tumor specimens included biopsy samples for definite diagnosis; thus, this may have biased the results of our study. However, it is difficult to absolutely perform surgical resection of primary tumors or metastatic sites. Second, there are no established methods for counting TILs in tumors and stroma. Our study analyzed TIL evaluation based on the approximate value of median counts. Considering previous studies, cut-off values using median counts may be useful. In the present study, the assessment of TILs was performed on the tumor specimens of biopsies and resections. The difference of cell counts between biopsies and resections was adjusted by the evaluation to normalize to 1000 cells. Therefore, we believe that the assessment for TILs displayed a consistent impact on prognostic value even if including surgical sample.

Finally, we didn’t compare the tumor immune environment between first-line and subsequent PD-1 blockade therapy. After repeated chemotherapy, the tumor immune environment may change; therefore, the prognostic role of TILs in the tumor and stroma may be different between patients with untreated and previously-treated NSCLC.

In conclusion, stromal CD4 TILs were significantly associated with predicting the outcome of first-line pembrolizumab treatment, especially in patients with non-AC and high PD-L1 expression. Although intratumoral CD8 TILs were useful in predicting OS after first-line pembrolizumab, stromal CD4 TILs and intratumoral CD8 TILs may have an overall same impact on the outcome after first-line pembrolizumab. Further studies are warranted to elucidate the prognostic significance of stromal CD4 and intratumoral CD8 TILs in patients who received ipilimumab plus nivolumab.

## Supplementary Information

**Additional file 1: Figure S1:** Numbers of intratumoral and stromal TILs according to PD-L1 expression. The numbers of CD4, CD8, Foxp3 and PD-1 TILs in the tumor and stroma were not significantly different based on PD-L1 expression of 1–49% and 50–100%.

**Additional file 2: Figure S2:** Numbers of intratumoral and stromal TILs according to tumor response. The numbers of CD4, CD8, Foxp3 and PD-1 TILs in the tumor and stroma were not significantly different based on PR and non-PR.

**Additional file 3: Table S1. **Patient’s characteristics according to the level of different TILs in tumor.

**Additional file 4: Table S2. **Patient’s characteristics according to the level of different TILs in stroma.

**Additional file 5: Table S3.** Correlation between intratumoral/stromal TILs (CD4, CD8, Foxp3, PD-1).

## Data Availability

The datasets generated during and/or analysed during the current study are available from the corresponding author on reasonable request.

## References

[1] Gadgeel SM, Stevenson JP, Langer CJ, Gandhi L, Borghaei H, Patnaik A (2018). Pembrolizumab and platinum-based chemotherapy as first-line therapy for advanced non-small-cell lung cancer: phase I cohorts from the KETNOTE-021 study. Lung Cancer.

[2] Hashemi S, Fransen MF, Niemeijer A, Taleb NB, Houda I, Veltman J (2021). Surprising impact of stromal TIL’s on immunotherapy efficacy in a real-world lung cancer study. Lung Cancer.

[3] Hu-Lieskovan S, Lisberg A, Zaretsky JM, Grogan TR, Rizvi H, Wells DK (2019). Tumor characteristics associated with benefit from pembrolizumab in advanced non-small cell lung cancer. Clin Cancer Res.

[4] Uryvaev A, Passhak M, Hershkovits D, Sabo E, Bar-Sela G (2018). The role of tumor-infiltrating lymphocytes (TILs) as a predictive biomarker of response to anti-PD-1 therapy in patients with metastatic non-small cell lung cancer or metastatic melanoma. Med Oncol.

[5] Kagamu H, Yamasaki S, Kitano S, Yamaguchi O, Mouri A, Shino A (2022). Discover of a new CD4^+^ T cell cluster that correlates PD-1 blockade efficacy. Cancer Res.

[6] Eisenhauer EA, Therasse P, Bogaerts J, Schwartz LH, Sargent D, Ford R (2009). New response evaluation criteria in solid tumour: revised RECIST guideline (version 1.1). Eur J Cancer.

[7] Halse H, Colebatch AJ, Petrone P, Henderson MA, Mills JK, Snow H (2018). Multiplex immunohistochemistry accurately defines the immune context of metastatic melanoma. Sci Reports.

[8] Geng Y, Shao Y, He W, Hu W, Xu Y, Chen J (2015). Prognostic role of tumor-infiltrating lymphocytes in lung cancer: a meta-analysis. Cell Physiol Biochem.

[9] Wei X, Gu K, Heng W (2021). T lymphocytes related biomarkers for predicting immunotherapy efficacy in non-small cell lung cancer (Review). Oncol Lett.

[10] Gataa I, Mezquita L, Rossoni C, Auclin E, Kossai M, Aboubakar F (2021). Tumour-infiltrating lymphocyte density is associated with favourable outcome in patients with advanced non-small cell lung cancer treated with immunotherapy. Eur J Cancer.

[11] Facchinetti F, Maio M, Perrone F, Tiseo M (2021). First-line immunotherapy in non-small cell lung cancer patients with poor performance status: a systematic review and meta-analysis. Transl Lung Cancer Res.

[12] Kano H, Ichihara E, Harada D, Inoue K, Kayatani H, Hosokawa S (2020). Utility of immune checkpoint inhibitors in non-small-cell lung cancer patients with poor performance status. Cancer Sci.

[13] Zeng DQ, Yu YF, Qu QY, Li XY, Zhong RZ, Xie CM (2016). Prognostic and predictive value of tumor-infiltrating lymphocytes for clinical therapeutic research in patients with n-small cell lung cancer. Oncotarget.

[14] Lin Z, Gu J, Cui X, Huang L, Li S, Feng J (2019). Deciphering microenvironment of NSCLC based on CD8+ TIL density and PD-1/PD-L1 expression. J Cancer.

[15] Fumet JD, Richard C, Ledys F, Klopfenstein Q, Joubert P, Routy B (2018). Prognostic and predictive role of CD8 and PD-L1 determination in lung tumor tissue of patients under anti-PD-1 therapy. Br J Cancer.

[16] Kagamu H, Kitano S, Yamaguchi O, Yoshimura K, Horimoto K, Kitazawa M (2020). CD4^+^ T-cell immunity in the peripheral blood correlates with response to anti-PD-1 therapy. Cancer Immunol Res.

[17] Inomata M, Kado T, Okazawa S, Okazawa S, Imanishi S, Taka C (2019). Peripheral PD-1-positive CD4 T-lymphocyte count can predict progression-free survival in patients with non-small cell lung cancer receiving immune checkpoint inhibitor. Anticancer Res.

[18] Niemeijer ALN, Sahba S, Smit EF, Lissenberg-Whitte BI, de Langen AJ, Thunnissen E (2020). Association of tumour and stroma PD-1, PD-L1, CD3, CD3, CD4 and CD8 expression with DCB and OS to nivolumab treatment in NSCLC patients pre-treated with chemotherapy. Br J Cancer.

[19] Soo RA, Chen Z, Teng RSY, Tan HL, Lacopetta B, Tai BC (2018). Prognostic significance of immune cells in non-small cell lung cancer: meta-analysis. Oncotarget.

