# Supplementary figures and images for: Prognostic significance of tumor infiltrating lymphocytes on first-line pembrolizumab efficacy in advanced non-small cell lung cancer
Source: Discov Oncol. 2023 Jan 20;14:6. doi: 10.1007/s12672-023-00615-4 (PMC9859977; doi:10.1007/s12672-023-00615-4)

## Slide 1
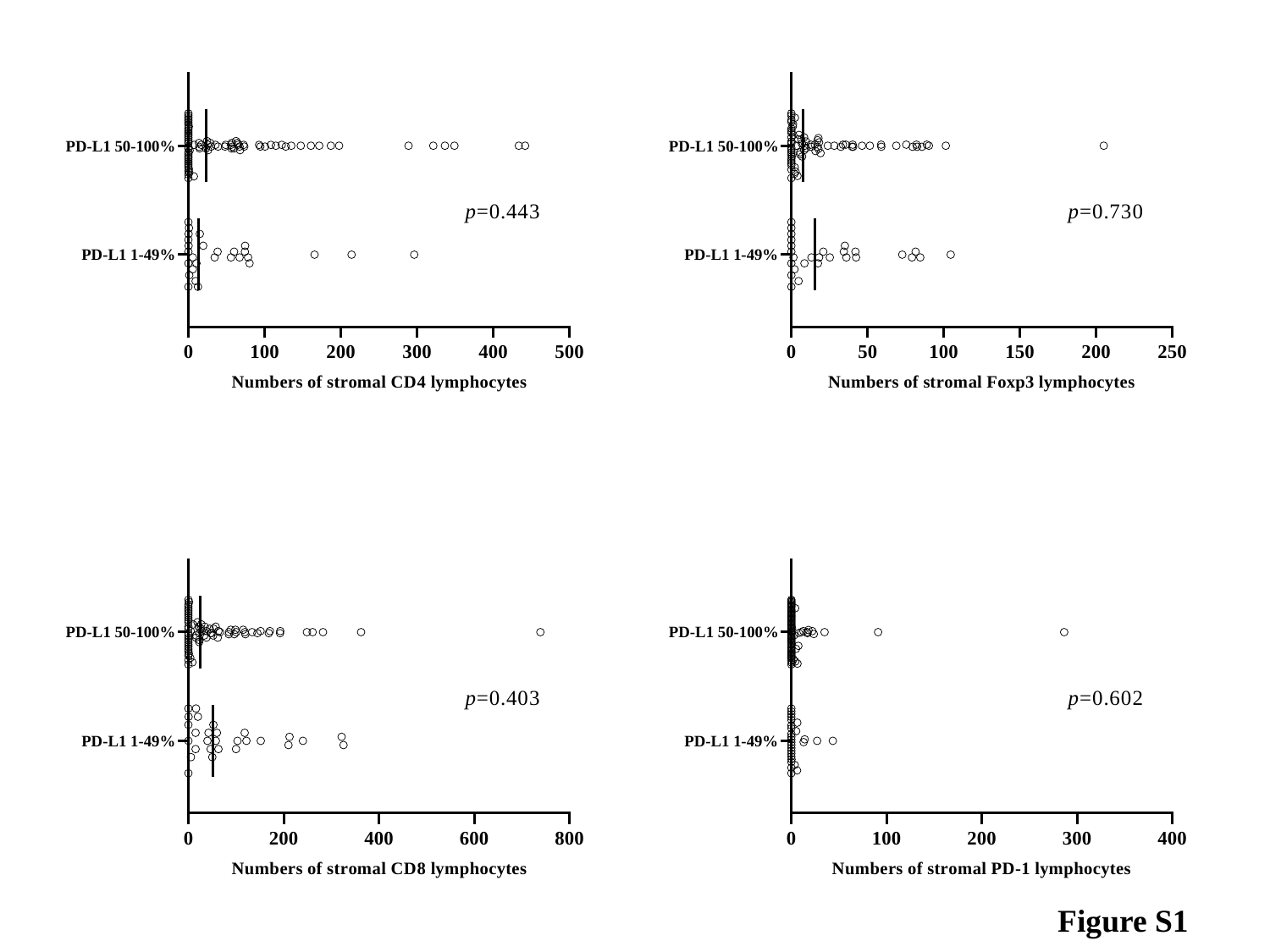

Figure S1

## Slide 2
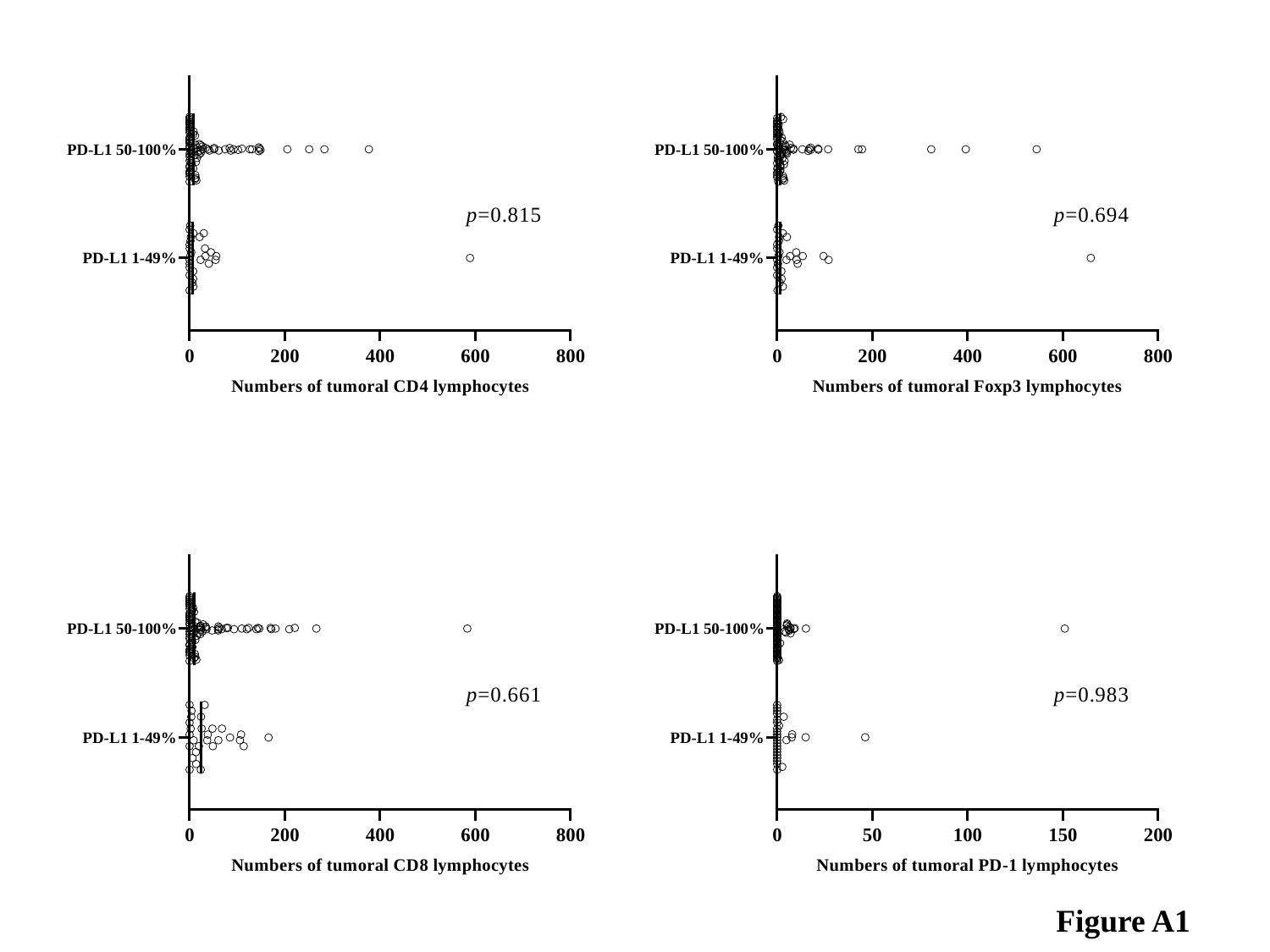

Figure A1

Supplement: Supplementary file 1 — Additional file 1: Figure S1: Numbers of intratumoral and stromal TILs according to PD-L1 expression. The numbers of CD4, CD8, Foxp3 and PD-1 TILs in the tumor and stroma were not significantly different based on PD-L1 expression of 1–49% and 50–100%. [file 12672_2023_615_MOESM1_ESM.pptx]

## Slide 1
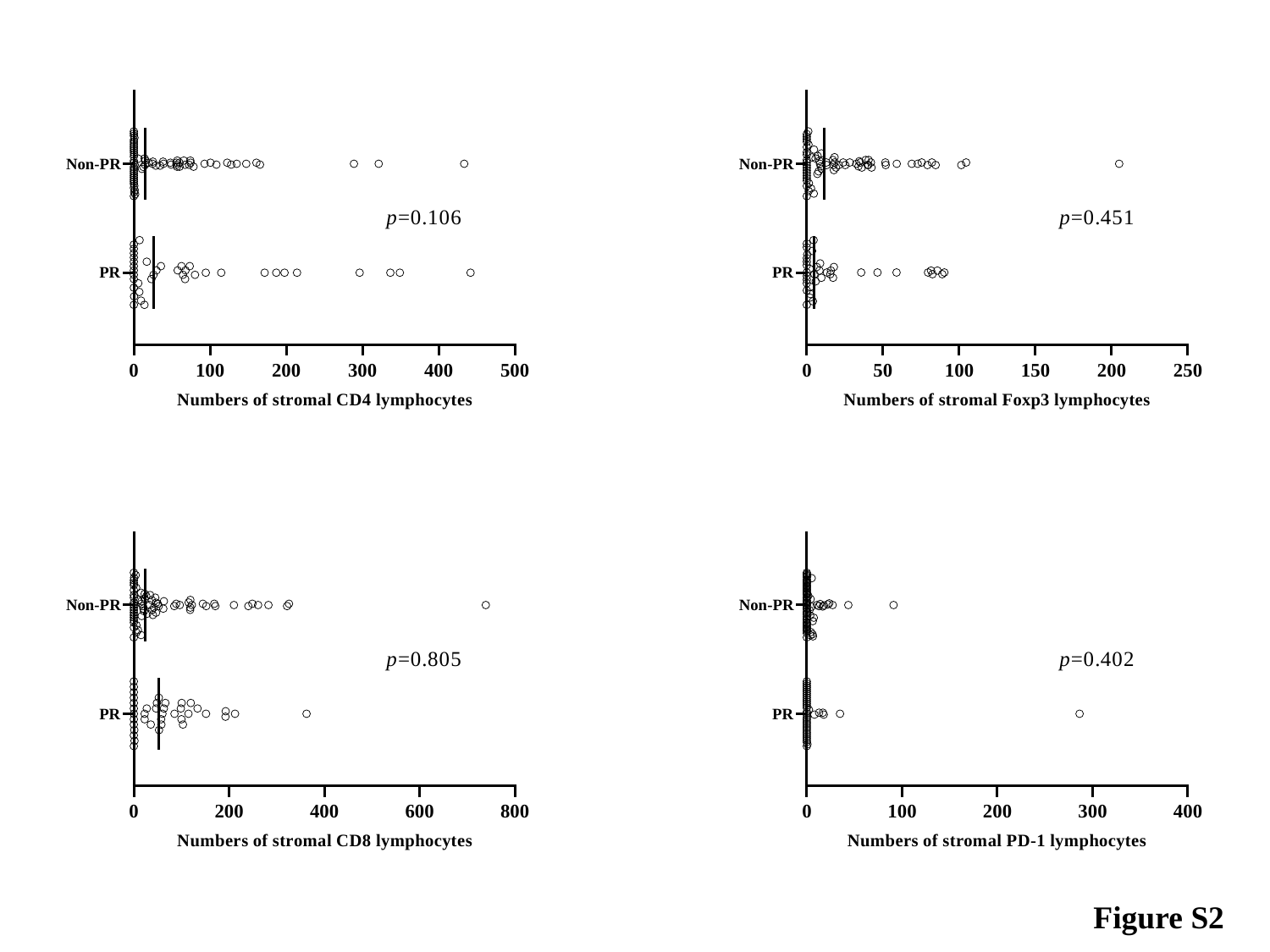

Figure S2

## Slide 2
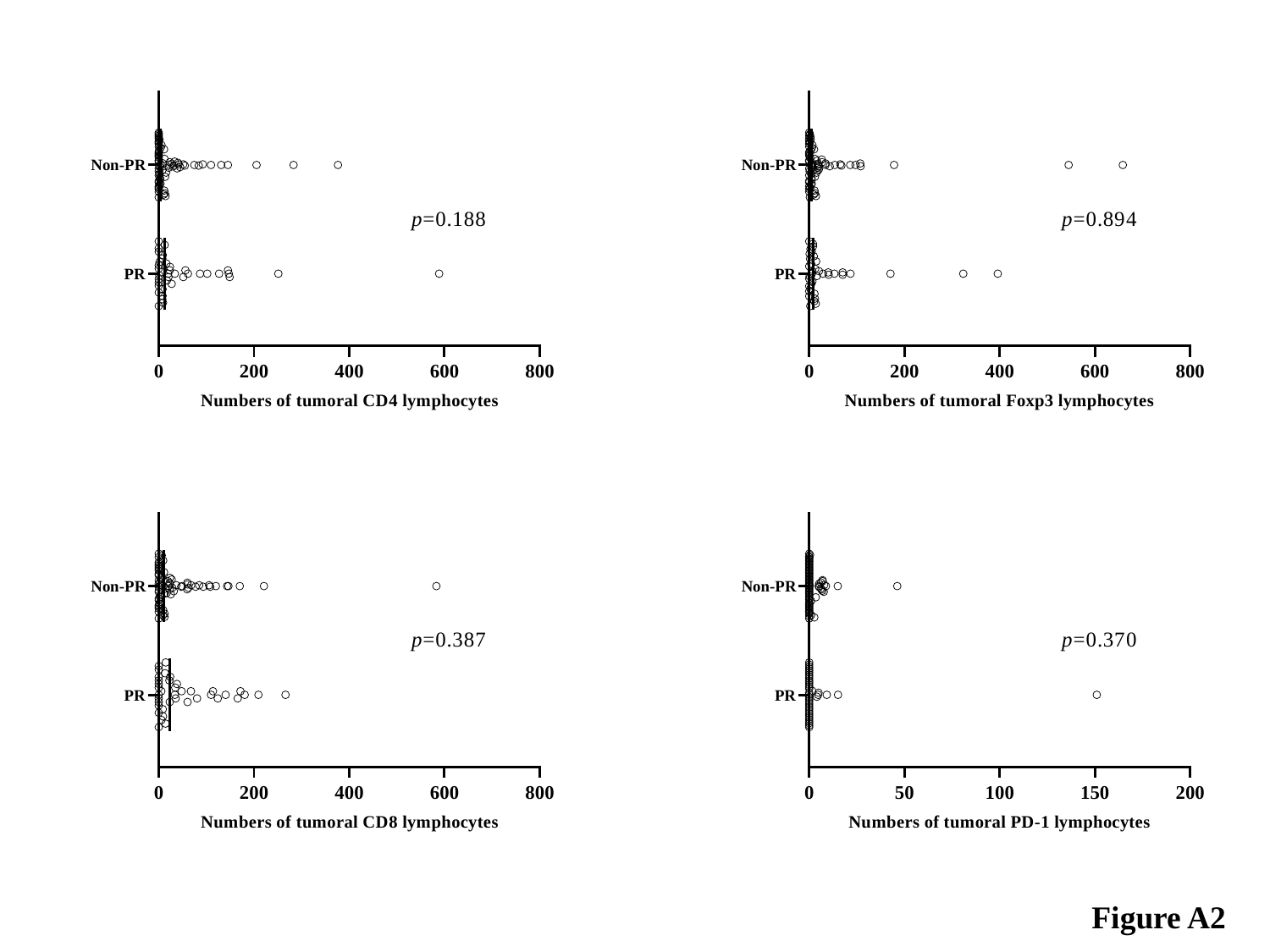

Figure A2

Supplement: Supplementary file 2 — Additional file 2: Figure S2: Numbers of intratumoral and stromal TILs according to tumor response. The numbers of CD4, CD8, Foxp3 and PD-1 TILs in the tumor and stroma were not significantly different based on PR and non-PR. [file 12672_2023_615_MOESM2_ESM.pptx]
