# Supplementary material for: Prognostic significance of tumor infiltrating lymphocytes on first-line pembrolizumab efficacy in advanced non-small cell lung cancer
Source: Discov Oncol. 2023 Jan 20;14:6. doi: 10.1007/s12672-023-00615-4 (PMC9859977; doi:10.1007/s12672-023-00615-4)
Supplement: Supplementary file 3 — Additional file 3: Table S1. Patient’s characteristics according to the level of different TILs in tumor. [file 12672_2023_615_MOESM3_ESM.docx]

**Table S1. Patient’s characteristics according to the level of different TILs in tumor**

| Variables | | CD4 TILs | | | CD8 TILs | | | Foxp3 TILs | | | PD-1 TILs | | | |
| --- | --- | --- | --- | --- | --- | --- | --- | --- | --- | --- | --- | --- | --- | --- |
|  |  | High  (n=50) | Low  (n=57) | *p*-value | High  (n=56) | Low  (n=51) | *p*-value | High  (n=45) | Low  (n=62) | *p*-value | High  (n=25) | Low  (n=82) | *p*-value |  |
| Age | <75 / ≥75 years | 32 / 18 | 32 / 25 | 0.435 | 36 / 20 | 28 / 23 | 0.332 | 26 / 19 | 38 / 24 | 0.841 | 19 / 6 | 45 / 37 | 0.066 |  |
| Gender | M / F | 42 / 8 | 49 / 8 | 0.792 | 47 / 9 | 44 / 7 | 0.791 | 36 / 9 | 55 / 7 | 0.274 | 22 / 3 | 69 / 13 | 0.758 |  |
| ECOG PS | 0-1 /2-3 | 39 / 11 | 43 / 14 | 0.821 | 44 / 12 | 38 / 13 | 0.653 | 38 / 7 | 46 / 16 | 0.239 | 19 / 6 | 63 / 19 | >0.999 |  |
| Smoking | Yes / No | 45 / 5 | 51 / 6 | >0.999 | 48 / 8 | 48 / 3 | >0.999 | 40 / 5 | 56 / 6 | >0.999 | 22 / 3 | 74 / 18 | 0.558 |  |
| Histology | AC / Non-AC | 23 / 27 | 29 / 28 | 0.699 | 29 / 27 | 23 / 28 | 0.562 | 32 / 13 | 20 / 42 | **<0.001** | 14 / 11 | 38 / 44 | 0.497 |  |
| Brain meta | Yes / No | 13 / 37 | 17 / 40 | 0.673 | 18 / 38 | 12 / 39 | 0.390 | 13 / 32 | 17 / 45 | >0.999 | 5 / 20 | 25 / 57 | 0.446 |  |
| Bone meta | Yes / No | 9 / 41 | 16 / 41 | 0.257 | 10 / 46 | 15 / 36 | 0.176 | 7 / 38 | 18 / 44 | 0.113 | 1 / 24 | 24 / 58 | **0.007** |  |
| Response | PR / Non-PR | 20 / 30 | 19 / 28 | >0.999 | 23 / 33 | 16 / 35 | 0.321 | 18 / 27 | 21 / 41 | 0.546 | 7 / 18 | 32 / 52 | 0.476 |  |
| PD-L1(%) | 1-49 / 50-100 | 10 / 40 | 16 / 41 | 0.372 | 16 / 40 | 10 / 41 | 0.367 | 10 / 35 | 16 / 46 | 0.819 | 8 / 17 | 18 / 64 | 0.301 |  |
| Prior RT | Yes / No | 19 / 31 | 19 / 38 | 0.687 | 25 / 31 | 13 / 48 | **0.009** | 16 / 29 | 22 / 40 | >0.999 | 12 / 13 | 26 / 56 | 0.156 |  |
| G3/4 irAE | Yes / No | 13 / 37 | 13 / 44 | 0.821 | 14 / 42 | 12 / 39 | >0.999 | 14 / 31 | 12 / 50 | 0.177 | 8 / 17 | 18 / 64 | 0.301 |  |
| Lymphocytes | High / Low | 26 / 24 | 27 / 30 | 0.700 | 31 / 25 | 22 / 29 | 0.247 | 21 / 24 | 32 / 30 | 0.696 | 15 / 10 | 38 / 44 | 0.260 |  |
| Albumin | High / Low | 26 / 24 | 29 / 28 | >0.999 | 31 / 25 | 24 / 27 | 0.441 | 23 / 22 | 32 / 30 | >0.999 | 13 / 12 | 42 / 40 | >0.999 |  |
| CRP | High / Low | 26 / 24 | 28 / 29 | 0.847 | 25 / 31 | 29 / 22 | 0.247 | 21 / 24 | 33 / 29 | 0.559 | 10 / 15 | 44 / 38 | 0.260 |  |

Abbreviations: ECOG, eastern cooperative oncology group; PS, performance status; PD-L1, programmed death ligand-1; PD-1, programmed death-1; irAE, immune-related adverse events; AC, adenocarcinoma; PR, partial response; CRP, C-reactive protein; meta, metastasis; Prior RT, radiation before initial pembrolizumab; TILs, tumor infiltrative lymphocytes.
