# Supplementary material for: Prognostic significance of tumor infiltrating lymphocytes on first-line pembrolizumab efficacy in advanced non-small cell lung cancer
Source: Discov Oncol. 2023 Jan 20;14:6. doi: 10.1007/s12672-023-00615-4 (PMC9859977; doi:10.1007/s12672-023-00615-4)
Supplement: Supplementary file 4 — Additional file 4: Table S2. Patient’s characteristics according to the level of different TILs in stroma. [file 12672_2023_615_MOESM4_ESM.docx]

**Table S2. Patient’s characteristics according to the level of different TILs in stroma**

| Variables | | CD4 TILs | | | CD8 TILs | | | Foxp3 TILs | | | PD-1 TILs | | | |
| --- | --- | --- | --- | --- | --- | --- | --- | --- | --- | --- | --- | --- | --- | --- |
|  |  | High  (n=51) | Low  (n=56) | *p*-value | High  (n=54) | Low  (n=53) | *p*-value | High  (n=49) | Low  (n=58) | *p*-value | High  (n=29) | Low  (n=78) | *p*-value |  |
| Age | <75 / ≥75 years | 31 / 20 | 33 / 23 | >0.999 | 32 / 22 | 32 / 21 | >0.999 | 30 / 19 | 34 / 24 | 0.844 | 20 / 9 | 44 / 34 | 0.273 |  |
| Gender | M / F | 42 / 8 | 48 / 8 | >0.999 | 45 / 9 | 46 / 9 | >0.999 | 43 / 6 | 48 / 10 | 0.589 | 25 / 4 | 66 / 12 | >0.999 |  |
| ECOG PS | 0-1 /2-3 | 46 / 5 | 36 / 20 | **0.002** | 42 / 12 | 40 / 13 | 0.822 | 36 / 13 | 46 / 12 | 0.500 | 24 / 5 | 56 / 22 | 0.320 |  |
| Smoking | Yes / No | 44 / 7 | 52 / 4 | 0.344 | 47 / 7 | 49 / 4 | 0.526 | 45 / 4 | 51 / 7 | 0.554 | 24 / 5 | 72 / 6 | 0.164 |  |
| Histology | AC / Non-AC | 28 / 23 | 24 / 32 | 0.248 | 27 / 27 | 25 / 28 | 0.847 | 29 / 20 | 23 / 35 | **0.049** | 17 / 12 | 35 / 43 | 0.276 |  |
| Brain meta | Yes / No | 17 / 34 | 13 / 43 | 0.285 | 18 / 36 | 12 / 41 | 0.282 | 12 / 37 | 18 / 40 | 0.520 | 8 / 21 | 22 / 56 | >0.999 |  |
| Bone meta | Yes / No | 4 / 47 | 21 / 35 | **<0.001** | 9 / 45 | 16 / 37 | 0.114 | 7 / 42 | 18 / 40 | 0.067 | 2 / 27 | 23 / 55 | **0.018** |  |
| Response | PR / Non-PR | 21 / 30 | 18 / 38 | 0.421 | 23 / 31 | 16 / 37 | 0.229 | 14 / 45 | 25 / 32 | **0.030** | 7 / 22 | 22 / 55 | 0.807 |  |
| PD-L1(%) | 1-49 / 50-100 | 10 / 41 | 16 / 40 | 0.367 | 16 / 38 | 10 / 43 | 0.260 | 15 / 44 | 11 / 37 | 0.823 | 8 / 21 | 18 / 60 | 0.620 |  |
| Prior RT | Yes / No | 23 / 28 | 15 / 41 | 0.068 | 22 / 32 | 16 / 37 | 0.313 | 25 / 24 | 13 / 45 | **0.002** | 14 / 15 | 24 / 54 | 0.113 |  |
| G3/4 irAE | Yes / No | 11 / 40 | 15 / 41 | 0.652 | 12 / 42 | 14 / 39 | 0.657 | 17 / 32 | 9 / 49 | **0.025** | 8 / 21 | 18 / 60 | 0.620 |  |
| Lymphocytes | High / Low | 29 / 22 | 24 / 32 | 0.177 | 28 / 26 | 25 / 28 | 0.700 | 25 / 24 | 28 / 30 | 0.847 | 16 / 13 | 37 / 41 | 0.519 |  |
| Albumin | High / Low | 30 / 21 | 25 / 31 | 0.176 | 30 / 24 | 25 / 28 | 0.441 | 26 / 23 | 29 / 29 | 0.846 | 18 / 11 | 37 / 41 | 0.198 |  |
| CRP | High / Low | 24 / 27 | 30 / 26 | 0.563 | 24 / 30 | 30 / 23 | 0.248 | 23 / 26 | 31 / 27 | 0.562 | 12 / 17 | 41 / 36 | 0.383 |  |

Abbreviations: ECOG, eastern cooperative oncology group; PS, performance status; PD-L1, programmed death ligand-1; PD-1, programmed death-1; irAE, immune-related adverse events; AC, adenocarcinoma; PR, partial response; CRP, C-reactive protein; meta, metastasis; Prior RT, radiation before initial pembrolizumab; TILs, tumor infiltrative lymphocytes.
