# Supplementary material for: Prognostic significance of tumor infiltrating lymphocytes on first-line pembrolizumab efficacy in advanced non-small cell lung cancer
Source: Discov Oncol. 2023 Jan 20;14:6. doi: 10.1007/s12672-023-00615-4 (PMC9859977; doi:10.1007/s12672-023-00615-4)
Supplement: Supplementary file 5 — Additional file 5: Table S3. Correlation between intratumoral/stromal TILs (CD4, CD8, Foxp3, PD-1). [file 12672_2023_615_MOESM5_ESM.docx]

**Table S3. Correlation between intratumoral/stromal TILs (CD4, CD8, Foxp3, PD-1)**

| Pearson γ  (*p*-value) | | Intratumoral sites | | | | Stromal sites | | | |
| --- | --- | --- | --- | --- | --- | --- | --- | --- | --- |
|  |  | **PD-1** | **CD4** | **CD8** | **Foxp3** | **PD-1** | **CD4** | **CD8** | **Foxp3** |
| Intratumoral sites | **PD-1** | NA | 0.017  (0.856) | 0.002  (0.981) | -0.039  (0.684) | 0.907  (**<0.001**) | 0407  (**<0.001**) | -0.016  (0.866) | 0.037  (0.702) |
|  | **CD4** | NA | NA | 0.247  (**0.009**) | 0.036  (0.703) | -0.009  (0.921) | 0.624  (**<0.001**) | 0.063  (0.511) | 0.021  (0.821) |
|  | **CD8** | NA | NA | NA | 0.139  (0.147) | -0.019  (0.843) | 0.171  (0.075) | 0.741  (**<0.001**) | 0.174  (0.070) |
|  | **Foxp3** | NA | NA | NA | NA | -0.039  (0.684) | 0.075  (0.437) | 0.216  (**0.023**) | 0.386  (**<0.001**) |
| Stromal sites | **PD-1** | NA | NA | NA | NA | NA | 0.407  (**<0.001**) | 0.017  (0.858) | 0.049  (0.611) |
|  | **CD4** | NA | NA | NA | NA | NA | NA | 0.209  (**0.029**) | 0.068  (0.480) |
|  | **CD8** | NA | NA | NA | NA | NA | NA | NA | 0.287  (**0.002**) |
|  | **Foxp3** | NA | NA | NA | NA | NA | NA | NA | NA |

Abbreviation: NA, not applicable.
